# A Case of Horner’s Syndrome Aiding the Diagnosis of Internal Carotid Artery Dissection (ICAD): A Life-Saving Twist of Fate

**DOI:** 10.7759/cureus.58367

**Published:** 2024-04-16

**Authors:** Deepaswi Bhavsar, Shreya Gandhi, Renu Magdum, Iqra Mushtaq, Nilesh Giri

**Affiliations:** 1 Department of Ophthalmology, Dr. D. Y. Patil Medical College, Hospital and Research Centre, Pune, IND

**Keywords:** ptosis, sympathetic involvement, cranial nerve paresis, carotid artery dissection, horner’s syndrome

## Abstract

A 59-year-old male patient came to the outpatient department with complaints of left-sided hemicranial headache with drooping of the left upper eyelid (UL) for three days associated with difficulty in swallowing and deviation of the tongue. The patient had a history of vigorous coughing for the past 15 days for which he did not take any medications. He was thoroughly evaluated in the outpatient department and diagnosed with Horner's syndrome. Acute Horner's syndrome with pain is nearly a hallmark of carotid dissection, and MRI of the brain and orbit was thus advised. On MRI, a hyperdense area was noted around the left internal carotid artery for which he was advised magnetic resonance angiography, which revealed internal carotid artery dissection (ICAD) of the left side. The patient was diagnosed with left-sided Horner's syndrome following left ICAD with involvement of the left hypoglossal nerve. He was started on antiplatelets and anticoagulants and closely followed up. Early diagnosis and prompt treatment were lifesaving for this patient.

## Introduction

Horner’s syndrome typically presents as ipsilateral miosis, ptosis, anhidrosis, loss of ciliospinal reflex, enophthalmos, and heterochromia iridium when the sympathetic innervation of these areas is disrupted. Internal carotid artery dissection (ICAD) is a rare entity yet one of the leading causes of cerebrovascular accidents in young populations approximately in the fourth to fifth decades of life, accounting for 10-20% of ischemic strokes [1]. The sequelae of dissection are pain, Horner’s syndrome, cranial nerve paralysis, or cerebral embolism. Potential precipitating causes are football, jogging, swimming, interventions like intubation, and others including coughing, vomiting, and sneezing [2]. The prevalence of early stroke recurrence has led many physicians to insist upon the utility of anticoagulants from presentation until three to six months after dissection which has been proven to be life-saving [2].

## Case presentation

A 59-year-old male patient came to the outpatient department with left-sided hemicranial headache with drooping of UL of the left eye for the past three days. The headache had no aggravating or relieving factors. He also complained of difficulty in swallowing and moving his tongue for three days. The patient gave a history of cold and vigorous cough for the past 15 days for which he did not take any medications. He was an avid trekker and a gym enthusiast. There was no history of trauma. He had no systemic illness like diabetes mellitus, hypertension, ischemic heart disease (IHD), or asthma, and was a non-alcoholic and non-smoker.

**Figure 1:**
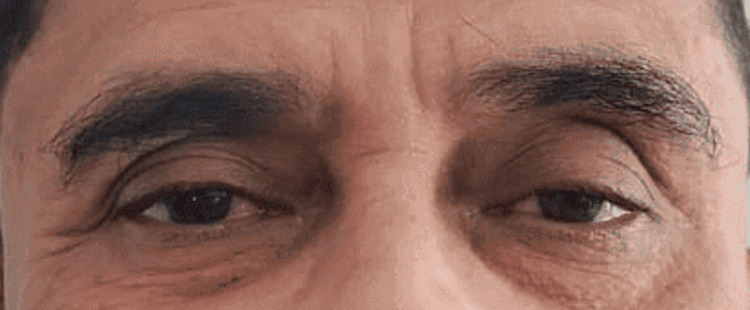
Left eye depicting mild ptosis

On ocular examination, the patient's best corrected visual acuity was 6/6 in both eyes with near and color vision within normal limits. On anterior segment examination, there was mild ptosis in the left eye. marginal reflex distance (MRD) 1 was 2 mm. MRD2 was normal. There was left-eye miosis. The rght-eye pupil was 4 mm and the left-eye pupil was 2 mm in size (Figure 1). Direct and consensual light reflexes were intact in both eyes. The rest of the anterior segment was within normal limits. Fundus examination of both eyes was within normal limits.

On systemic examination, there was a deviation of the tongue to the left side with difficulty in swallowing, suggestive of hypoglossal nerve involvement. Other cranial nerves were checked and were within normal limits. During a neuro-ophthalmologic evaluation, ophthalmoscopic and visual-field examinations did not show any more abnormalities. The patient was identified as having left-sided Horner's syndrome with ipsilateral involvement of the 12th cranial nerve. A dissection of the carotid artery was then one of the most crucial factors to take into consideration since it can compress the nearby ascending sympathetic plexus or cranial nerve XII at the base of the skull which might result in tongue deviation and ipsilateral Horner syndrome. So, the patient was suggested an MRI brain and orbit.

**Figure 2:**
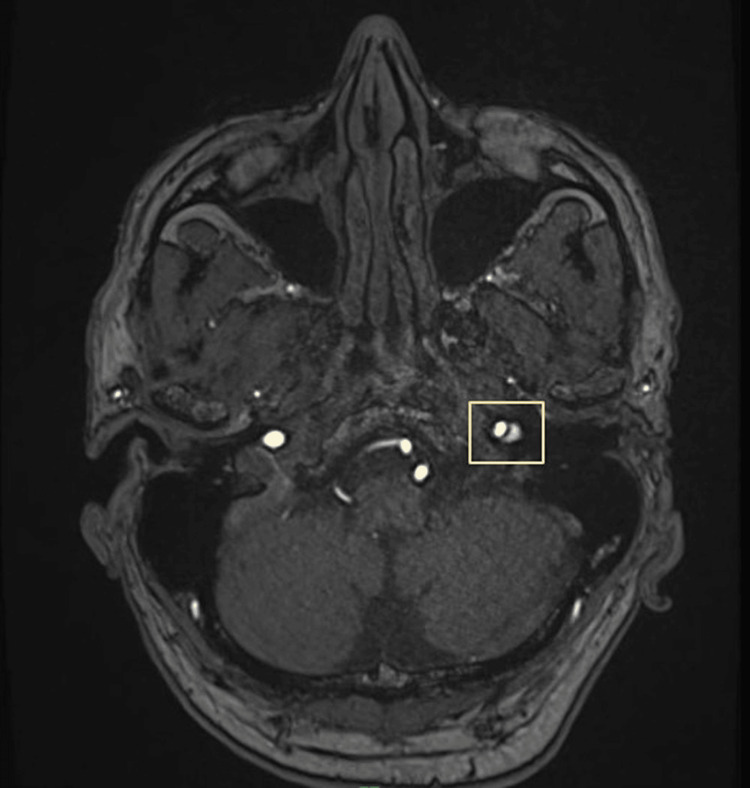
MRA transverse section depicting thin hypointense intimal flap within the left ICA with eccentric false lumen compressing the true lumen suggestive of dissection of the left internal carotid artery. MRA: magnetic resonance angiography; ICA: internal carotid artery

**Figure 3:**
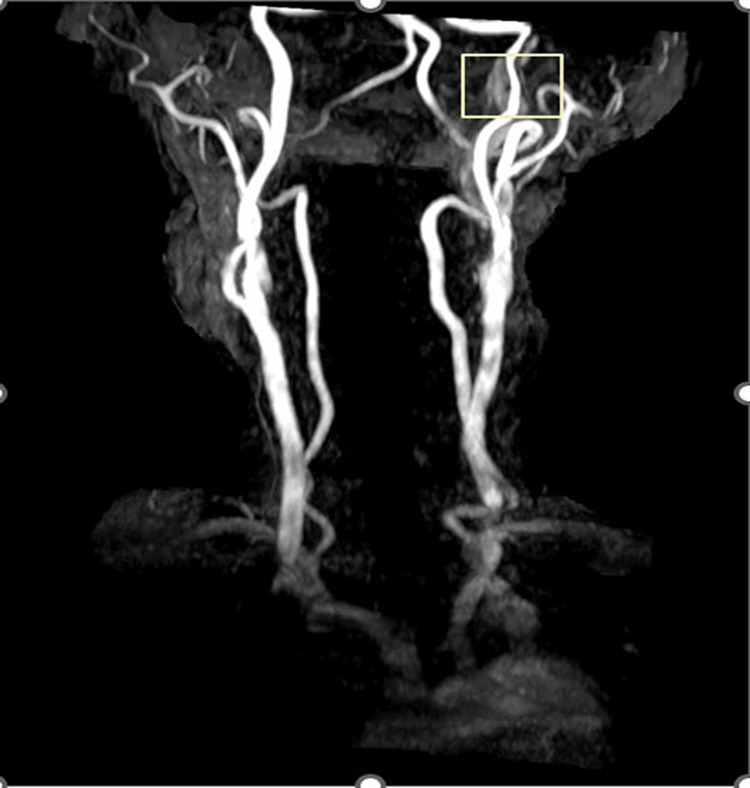
MRA showing luminal narrowing in left internal carotid artery dissection. MRA: magnetic resonance angiography

MRI showed subtle intramural soft-tissue thickening of the left-sided internal carotid artery (ICA) proximal to the petrous portion for which an MRI of the brain with neck angiography was suggested and performed. The diagnosis was confirmed on magnetic resonance angiogram (MRA), which revealed dissection of the left cervical ICA 12 mm after carotid bulb with a long segment intimal flap and intramural hematoma extending superiorly till petrous segment. The remaining intracranial segment of the ICA was normal (Figures 2, 3).

**Figure 4:**
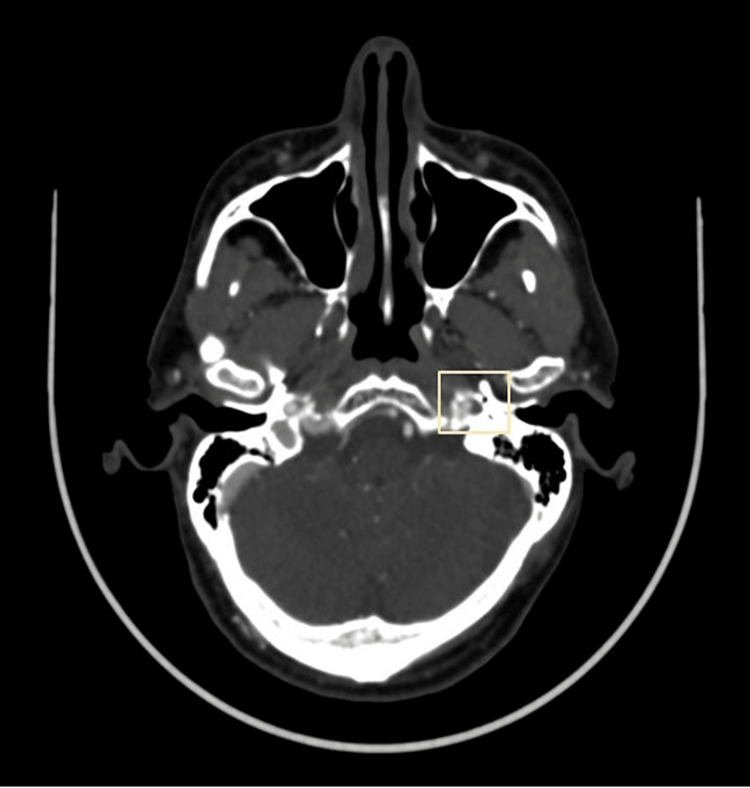
CT angiography showing a narrow opacified true lumen with a crescent sign suggestive of false lumen due to dissection.

Further, the patient underwent CT angiography to monitor the blood flow reaching the brain through the affected ICA and also to decide whether the patient would need surgical intervention. CT angiography revealed a narrow opacified lumen at the cervical segment of the left ICA. A crescent-shaped hypodense material showing contrast non-opacification was noted in the cervical segment of the left ICA, suggestive of false lumen due to dissection known as the crescent sign (Figure 4).

The patient was started on Injection enoxaparin 0.6 ml subcutaneously twice daily for seven days followed by oral anticoagulant and antiplatelet medications which included tablet aspirin 150 mg once daily, tablet atorvastatin 40 mg once daily and tablet clopidogrel 75 mg once daily for a month and advised a close follow-up. The patient was counseled to immediately stop neck exercises and hyperextensions,avoid driving, gyming, and all the strenuous activities which could potentially increase the intra-abdominal pressure, thus further damaging the ICA. He was instructed to watch for any tingling, numbness, or giddiness. Also, he was advised a repeat CT angiography to monitor the blood flow after one month.

He was followed up after a month. All symptoms had drastically improved. There was no headache. Left eye ptosis had fully resolved; however, miosis was still present. There was no deviation of tongue and difficulty in swallowing had significantly reduced. The patient was on a close, timely follow-up, and on continuous oral anticoagulants and antiplatelets.

## Discussion

The sympathetic nervous system, which includes the dilator pupillae, Muller's muscle of the eyelid, and the sweat glands on the same side of the forehead, is responsible for ipsilateral mydriasis, proper lid position, and ipsilateral sweating of the forehead. Due to a disturbance in the sympathetic nerve supply, Horner's syndrome is an uncommon illness that is traditionally characterized by partial ptosis, miosis, and ipsilateral absence of sweating [1]. Although there have sporadically been congenital occurrences, it is primarily acquired. ICAD can cause unilateral headaches, Horner's syndrome, and paralysis of the cranial nerve as clinical symptoms. Horner's syndrome develops when the sympathetic fibers in the carotid wall are compressed or have less perfusion [3-5].

A few typical causes of Horner's syndrome include multiple sclerosis, cervical rib injuries, cerebrovascular accidents, and iatrogenic causes, depending on the involvement of first, second, or third-order neurons. Third-order neurons are impacted by carotico-cavernous fistula, ICAD, or aneurysm, among other conditions, and are situated close to the ICA and cavernous sinus. The most frequently afflicted cranial nerve is the XII, which is followed by the involvement of the IX, X, XI, and V nerves. One likely cause of the nerve palsy is a compromised vasa nervorum; direct compression by a mural hematoma is another possibility [4].

When there is a partial tear in the artery's tunica intima, the carotid artery becomes dissected. A hematoma that originates intramurally in the tunica media causes lumen stenosis. Approximately 90% of extracranial ICA dissections occur, which is far more common than intracranial [6]. ICAD occurs in 2.5-3.0 out of every 100,000 people. Although the occurrence ratio in males and females is equal, females are often younger at the time of dissection. In middle-aged patients, dissection of the carotid and vertebral arteries is a major risk factor for ischemic stroke, accounting for 10-25% of cases in this age range [3]. The prognosis for ICAD is highly unforeseeable.

Horner syndrome was hardly associated with ICA dissection in the past. After examining 450 cases of Horner's disease, one study found that the most frequent causes were tumors (13%) and cluster headaches (12%). Carotid dissection is now more commonly recognized as a cause of the syndrome with the emergence of MRI. In a prospective investigation by Digre et al., MRI was performed on 33 individuals who had been diagnosed with Horner syndrome, and angiograms verified the carotid artery dissection in 15% of the patients [4].

Anticoagulants may prevent embolism from a newly formed thrombus, but they are more perilous than antiplatelets and can cause intramural hemorrhage to spread, which is seen in approximately one-third of patients based on MRI. In the event that anticoagulants are ineffective or contraindicated, the patient's luminal diameter can be promptly restored through either routine monitoring or surgical intervention using intravascular stents [2].

## Conclusions

A life-threatening complication was avoided as ICAD was diagnosed on the basis of ocular manifestations of Horner’s syndrome in this patient. In order to prevent any ischemia insults to the brain or ocular tissues, prompt attention and referral for urgent imaging examinations using a mix of anticoagulants and antiplatelets were essential in this situation. When such patients are promptly and appropriately managed, most of them have extremely good prognoses and outcomes, which lowers morbidity and death.

Thus, from our inference, carotid artery dissection should be diagnosed and treated as soon as possible as it could be life-saving and help avoid cerebral ischemia and the subsequent development of systemic problems, which could result in morbidity and death.
